# The role of ESAT-6 in tuberculosis immunopathology

**DOI:** 10.3389/fimmu.2024.1383098

**Published:** 2024-04-03

**Authors:** Beatriz B. S. Passos, Mariana Araújo-Pereira, Caian L. Vinhaes, Eduardo P. Amaral, Bruno B. Andrade

**Affiliations:** ^1^ Curso de Medicina, Universidade Salvador, Salvador, Brazil; ^2^ Multinational Organization Network Sponsoring Translational and Epidemiological Research (MONSTER) Initiative, Salvador, Brazil; ^3^ Instituto de Pesquisa Clínica e Translacional, Faculdade Zarns, Clariens Educação, Salvador, Brazil; ^4^ Laboratório de Pesquisa Clínica e Translacional, Instituto Gonçalo Moniz, Fundação Oswaldo Cruz, Salvador, Brazil; ^5^ Programa de Pós-Graduação em Medicina e Saúde Humana, Escola Bahiana de Medicina e Saúde Pública (EBMSP), Salvador, Brazil; ^6^ Departamento de Infectologia, Hospital das Clínicas, Faculdade de Medicina da Universidade de São Paulo, São Paulo, Brazil; ^7^ Inflammation and Innate Immunity Unit, Laboratory of Clinical Immunology & Microbiology, National Institute of Allergy and Infectious Diseases, National Institutes of Health, Bethesda, MD, United States

**Keywords:** tuberculosis, ESAT-6, immune activation, cell death, immunopathology

## Abstract

Despite major global efforts to eliminate tuberculosis, which is caused by *Mycobacterium tuberculosis* (Mtb), this disease remains as a major plague of humanity. Several factors associated with the host and Mtb interaction favor the infection establishment and/or determine disease progression. The Early Secreted Antigenic Target 6 kDa (ESAT-6) is one of the most important and well-studied mycobacterial virulence factors. This molecule has been described to play an important role in the development of tuberculosis-associated pathology by subverting crucial components of the host immune responses. This review highlights the main effector mechanisms by which ESAT-6 modulates the immune system, directly impacting cell fate and disease progression.

## 1 Introduction

Tuberculosis (TB) is a leading cause of death due to a single infectious agent (1). The disease is caused by infection with *Mycobacterium tuberculosis* (Mtb), which is thought to spread through aerosolized particles originating from people with active pulmonary TB. Intriguingly, many individuals are infected but do not display any symptoms. Currently, the World Health Organization estimated that ¼ of the world’s population has been exposed to this pathogen (1). The factors determining the outcome of Mtb infection include several aspects related to the interaction between the host and the pathogen (2). After the first contact with Mtb, there is a robust induction of the many components of the immune response against Mtb, comprising activation of both innate and adaptive immune responses, that will determine the clinical outcome after the exposure, ranging from asymptomatic Mtb elimination to active diseases with a range of clinical manifestations. Nevertheless, the mycobacterium has evolved a variety of strategies to subvert the inflammatory milieu, attempting to thrive. In the active disease states, the strategies of subversion of the Mtb leads to a chronic inflammation process, and a cascade of immune activation aiming at the Mtb elimination. However, this process often results in collateral tissue damage. The mechanisms leading to such scenario are intricate and multifactorial, ranging from simply accumulation of free radicals, as observed in cells and tissues during oxidative stress induced by active infection, to regulation of pathways that dictates cell death modalities.

Successful pathogens employ evolutionary conserved strategies to evade the host immune response, allowing their growth and dissemination. Bacteria from the *Mycobacterium tuberculosis complex* (MTBC) perform such evasion by a variety of mechanisms, including the utilization of protein secretion systems that disrupt the physiology and metabolism of infected cells (2). Mtb possesses several virulence factors, encompassing cell surface components, enzymes involved in cellular metabolism, and transcriptional regulators (3). A large body of evidence indicates that Mtb exerts several of its pathogenic effects through secretion of a unique protein named 6kDa early secretory antigenic target (ESAT-6) (4). Hereafter, we will dissect historical, molecular, and biological features of such protein and its importance in TB pathogenesis, to propose new lines of study based on current knowledge.

## 2 The origin of ESAT-6: the revolution of virulence tags

Discovered in the mid-1990s, ESAT-6 was first described as a protein secreted in short-term Mtb culture and capable of inducing robust immune activation (5). This protein is secreted by the ESAT-6 secretion system-1 (ESX-1), also kwon as the type VII secretion system (T7SS) (6–8). ESX-1 apparatus is induced by a genetic locus known as difference region (RD) 1, a DNA segment that encodes nine genes (Rv3871 to Rv3879c) (6, 9). The primary function of this molecular complex is to release a set of effector proteins, including ESAT-6, that help the pathogen to thrive through manipulation of immune responses. The RV3875 gene encodes the ESAT-6 (EsxA), while Rv3874 encodes the EsxB (culture filtrate protein 10, CFP-10) (9, 10). These proteins form highly immunogenic heterodimers that have been shown to promote bacterial virulence (6). Supporting the involvement of the RD1 locus in mycobacterial pathogenicity, the protein complex associated with RD1 is found in virulent strains of Mtb and *Mycobacterium bovis* (*M. bovis*), but is absent in avirulent Bacillus Calmette-Guérin (BCG) substrains, which are the live attenuated vaccine form of M. bovis (6, 11). The impact of RD1 on bacterial virulence was evaluated in H37Rv Mtb infected mice, a virulent strain, H37Rv:ΔRD1 (H37Rv strain lacking RD1 expression), and BCG-Russia strain (12). Lungs of H37Rv infected mice exhibited diffuse parenchymal inflammation, which was not observed in the lungs of mice infected with H37Rv:ΔRD1 and BCG-Russia strains (12). After RD1 locus introduction, was evidenced an extensive pulmonary tissue damage, restoring the virulence. This finding confirms the crucial role of RD1 in governing Mtb pathogenicity (13) raising suspicions about the role of ESAT-6 in inducing necrosis (13, 14).

**Figure 1:**
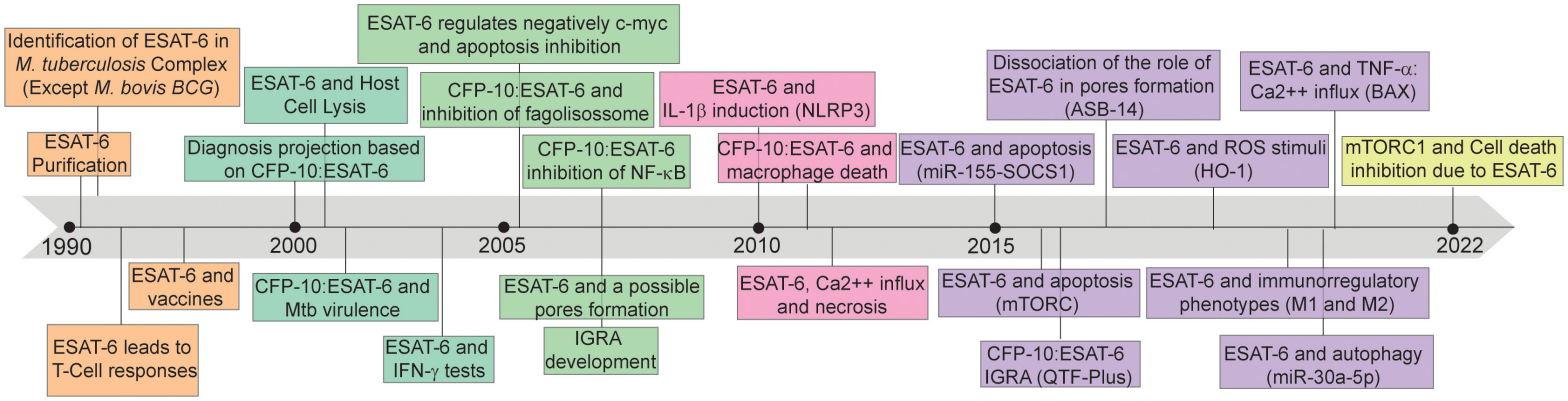
Timeline of discoveries associated with 6kDa early secretory antigenic target (ESAT-6). ESAT-6: 6kDa early secretory antigenic target; M. bovis BCG: Mycobacterium bovis Bacillus Calmette-Guérin; T-Cell: T lymphocytes; CFP-10: 10-kDa culture filtrate protein; Mtb: Mycobacterium tuberculosis; IFN-γ: Interferon-gamma; c-myc: MYC proto-oncogene; NF-K β: nuclear factor-kappa β; IGRA: Interferon-γ release assays; IL-1β: interleukin-1β; NLRP3: NLR Family Pyrin Domain Containing 3; Ca2++: calcium ion; miR-155: microRNA 155; SOCS1: suppressor of cytokine signaling 1; mTORC: mechanistic target of rapamycin kinase complex; QTF-Plus: QuantiFERON-TB Gold Plus; ROS: Reactive oxygen species; HO-1: Heme oxygenase 1; M1: Macrophages 1; M2: Macrophages 2; miR-30a-5p: microRNA 30a-5p; TNF- α: tumor necrosis factor alpha; BAX: BCL2 associated X; mTORC1: mammalian target of rapamycin complex 1.

Therefore, the interaction between ESAT-6 and the immune system plays a critical role in TB immunopathogenesis. By inducing immune activation, ESAT-6 can shape the host immune response, achieving the Mtb proliferation and dissemination after tissue damage mediated by the host inflammatory activation. The protein’s ability to trigger immune responses underscore its importance as a key virulence factor in Mtb infection (15). Understanding the mechanisms underlying ESAT-6-mediated immune activation is of paramount importance. ESAT-6 can modulate host immune responses through various pathways, including cytokine production, cell signaling, and immune cell recruitment (15). However, ESAT-6 has been found to be assigned with T cell epitopes recognized by major histocompatibility complex (MHC) class II, as well as epitopes recognized by B lymphocytes and antibodies (16). This pleiotropic behavior allowed the potential of ESAT-6 as a vaccine subunit to be explored (17), inspiring subsequent TB diagnostic advancements (18) and biomarker for disease severity (15, 19). The evolution of the knowledge about this protein and its impact on disease is depicted in **Figure 1**.

## 3 Effects of ESAT-6 on mycobacterial virulence: modulating host cell response

### 3.1 Receptors activation

**Figure 2:**
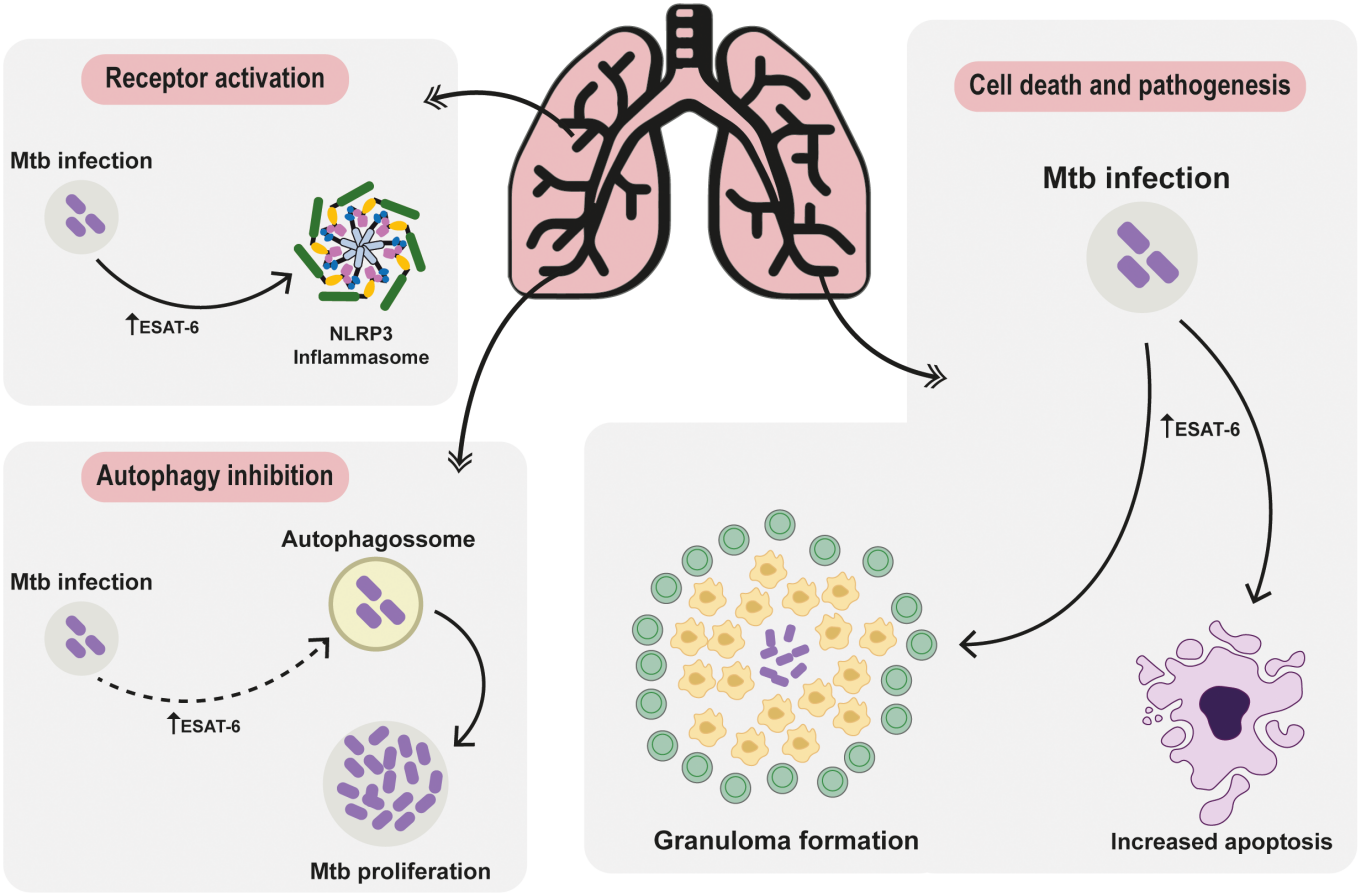
Deciphering ESAT-6: Unraveling Protein-Mediated Pathways in Mycobacterium tuberculosis Pathogenesis. The ESAT-6 protein intricately modulates receptor activation pathways, steering cellular autophagy regulation towards cell demise and pathogenesis. Post Mtb invasion, elevated ESAT-6 triggers NLRP3 inflammasome activation, pivotal IL-18 and IL-1β maturation, exacerbating pulmonary disease severity and hampering treatment responses. Following invasion, cells often activate autophagy. Upon thwarting autophagy to fuel pathogen growth, the immune defense transitions to apoptosis and necrosis, tightly regulated by ESAT-6. The involvement of ESAT-6 in necrosis weaves a complex narrative, fostering neutrophil-driven NETosis necrosis. This visual portrayal unveils ESAT-6’s intricate symphony, providing a fresh perspective on tuberculosis immune maneuvers and sparking novel avenues for therapeutic exploration.

The front line of host defense against Mtb is mediated by the innate immunity (20). Activation of receptors, particularly Toll-like receptors (TLRs), plays a crucial role in initiating the immune response against Mtb infection, that will determine the future outcomes, ranging from completely asymptomatic elimination of Mtb, to active infection, with several of clinical manifestation linked to the inflammatory activation. Upon reaching the respiratory tract and infecting alveolar macrophages, Mtb induces a cascade of signals, which subsequently triggers the recruitment and influx of other immune cells, including neutrophils and granulocytes (21). This orchestrated immune response sets the stage for the activation of TLRs, which recognize specific pathogen-associated molecular patterns (PAMPs) produced by Mtb, thereby triggering a robust immune reaction to combat the infection (21). The recognition of PAMPs during infection and activation of TLRs result in the induction of distinct intracellular signaling cascades, which in turn favor the production and secretion of inflammatory mediators critical for the host microbial response against the pathogens (22), as represented in **Figure 2**.

In Mtb infection, TLRs appear to be among the first to be stimulated upon specific PAMPs such as ESAT-6, involved in induction of many cellular responses and, consequently, being potential targets for regulation of antigen presentation by Mtb (23). The activation of TLRs can trigger downstream signaling pathways, leading to distinct outcomes depending on their association with MyD88, a critical adaptor molecule in the TLR signaling cascade (24). In MyD88-dependent pathways, TLR activation induces the production of pro-inflammatory cytokines and chemokines, immune cell recruitment, and upregulation of co-stimulatory molecules, facilitating antigen presentation to T cells and promoting the adaptive immune response against Mtb (25). Additionally, TLRs can activate MyD88-independent pathways, leading to interferon regulatory factor (IRF) activation and type I interferon (IFN) production, such as IFN-a and IFN-β (26). However, it has also been described that IFN- β activation by Mtb is independent of TLR signaling (27). The connection between ESX-1 (which main substrate is the ESAT-6) and the production of the type I IFN response has already been identified in previous studies (28, 29). Type I IFNs are strongly associated with increased susceptibility to pathogen-mediated cell death, favoring mycobacterial replication, tissue damage and Mtb dissemination (29, 30).

The expression of ESAT-6 by Mtb disrupts innate immune responses to infection by inhibiting TLR signaling pathways (31). The inhibitory effect mediated by TLR2 requires the presence of the six carboxy-terminal amino acid residues of ESAT-6 (31). This inhibition, facilitated by ESAT-6 binding to TLR2, requires the kinase Akt and prevents interaction between the adaptor MyD88 and downstream kinase interleukin-1 receptor-associated kinase (IRAK) 4 (4). The final consequence of this pathway is the blockage of Nuclear Factor kappa B (NF-κB) transcription and IRFs (31). Interestingly, while genetic depletion of ESAT-6 diminishes IFN-β expression, leading to decreased anti-mycobacterial defenses, stimulation of murine macrophages with ESAT-6 induces elevated IFN-β mRNA expression in a dose-dependent manner, dependent on TLR4 and TLR2/4 signaling (29). These findings underscore the critical role of ESAT-6 in modulating MyD88-dependent TLR signaling and suggest the potential use of mimetic inhibitory peptides to regulate innate immune responses when prolonged TLR signaling is detrimental (31).

TLR activation can be pleiotropic, inducing both harmful IFN-β and pro-inflammatory cytokines essential for host defense against Mtb infection, such as IL-1β and IL-18 (30). These cytokines are generated as inactive pro-forms that require caspase-1 activity to be cleaved into mature and bioactive form (32), mediating the activation of the inflammasome. ESAT-6 is the main Mtb PAMP that activates the NLRP3 inflammasome (33). Upon a secondary stimulus (34), NLRP3 oligomerizes and recruits pro-caspase-1 (35), cleaving pro-IL-1β and pro-IL-18 into their mature forms (36). In Mtb infection, ESAT-6 expression inhibits autophagy, resulting in damaged mitochondria accumulation and NLRP3 inflammasome activation (33). Additionally, it triggers the release of cathepsin B from lysosomes, which facilitates the secretion of mature IL-1β (37–39). IL-1β plays a crucial role in innate responses during TB infection, demonstrating direct antimicrobial potential in murine macrophages and human monocyte-derived macrophages (40). Despite its protective effects, since TB is an immunopathogenic diseases, high IL-1β expression genotypes have been associated to active TB, pulmonary disease severity, and unfavorable treatment outcomes (41).

### 3.2 Autophagy regulation and pore formation

Autophagy is a homeostatic process that contributes to the degradation and recycling of cellular components (42). Physiologically, autophagic organelles originating from the endoplasmic reticulum form the autophagosome through the activation of three major protein complexes: (i) Unc-51-like kinase (ULK) 1, (ii) Vacuolar Protein Sorting (VPS) 34 and (iii) the protein complex formed by the ATG16L1 – ATG5 – ATG12 conjugation (43). Thus, this complex works in a similar way to the E3 ubiquitin ligases, facilitating the binding of the ubiquitin-like ATG8 family. These proteins are essential for cargo recognition, closing autophagosomes and promoting their fusion with lysosomes (43). Mtb employs several strategies to subvert intracellular control mechanisms, including blocking phagosome-lysosome fusion through the ESAT-6 antigen (44–47). The ability to block the fusion of the phagosome with the lysosome, bypass the innate immune responses of the host, creating a favorable environment for their dissemination. This evasion strategy enables Mtb to replicate within early phagosomes (48). ESAT-6 blocks autophagy by activating the mammalian Target of Rapamycin (mTOR), a kinase that regulates changes related to cell growth, proliferation, and survival (49). The mTOR forms a complex subdivided into mTOR Complex 1 (responding to 4E-BP1 and S6K) and mTOR Complex 2 (which phosphorylates the C-terminus of some AGC kinases such as Akt and SGK) and suppression of mTORC1 positively regulates autophagy (50). ESAT-6-treated macrophages exhibited increased levels of the protein called microtubule-associated protein 1 light chain 3 (LC3), that plays a key role in the formation of autophagosomes, indicating autophagosome formation (49). Expanding beyond this pathway, ESAT-6 has demonstrated its influence on the intracellular survival pathway regulated by microRNA-30a (Mir-30a), which branches into two subdivisions: microRNA-30a-3p (Mir-30a-3p), known as an autophagic inhibitor, and microRNA-30a-5p (Mir-30a-5p), recognized as an autophagic promoter (51). Notably, ESAT-6 was observed exerting negative regulation on Mir-30a-5p, affecting the cellular autophagic processes. This interruption of a crucial homeostatic mechanism such as autophagy underscores ESAT-6’s role in facilitating the growth of Mtb and exacerbating the progression of the disease (51).

Additionally, utilizing the ESX-1 secretion system, some Mtb pathogens escape into the cytosol by releasing ESAT-6, which compromises the integrity of the phagosomal membrane (52). Certain mycobacteria possess the capability to translocate from the phagolysosomal compartment to the host cell’s cytosol through pore formation, a virulence linked to the RD1 gene (52). This disruption facilitates the exit of Mtb bacilli or their PAMPs from the vesicle, triggering innate sensors such as NLRP3 and AIM2. These sensors initiate the formation of inflammasomes, essential for IL-1β processing through caspase-1 activation (53, 54). Intriguingly, the specific presence of ESAT-6 correlates with the extent of tissue damage, severity, and the spread of infections, not only within the MTBC. The secretion of ESAT-6, also observed in *Mycobacterium marinum* (*M. marinum*) through the ESX-1 system, has been associated with clinical features akin Mtb (55) and pore formation (54, 56), aligning with previous research suggesting that ESAT-6’s structural alteration in an acidic medium prompts membrane vesicle leakage in Mtb strains (57).

However, recent evidence has cast doubt on the role of ESAT-6 as a pore-forming protein (58). Although previous research has implicated ESAT-6 in membrane lysis or pore formation (52, 54, 57, 59, 60), this hypothesis has been called into question due to potential detergent contamination (58). Instead, recent evidence suggests a form of contact-induced membrane rupture mediated by ESX-1 activity (53, 58). Hemolytic activity assays without ASB-14 confirmed the absence of lysis, suggesting that ASB-14 modifies ESAT-6 structure, activating its lytic activity (58). This damage may be facilitated by ESX-1-dependent factors in the bacterial capsule, or other components affected by detergent presence. Mtb cultured without detergent exhibited increased membrane-damaging activity, inflammasome activation, and cell death (53). Remarkably, ESX-1 displays bidirectional membrane-damaging activity, affecting both sides of the plasma membrane (PM) (53).

The inhibition of autophagy, maintaining the Mtb burden, and the membrane damage, leading to increases in PAMPs and direct Mtb trigger to immune sensor, generates a continuous process of inflammatory activation that contributes to tissue damage and Mtb dissemination.

### 3.3 The adaptive immunity and vaccines

Both the innate and adaptive responses are essential for protective immunity against Mtb (22). ESAT-6 activates receptors, promoting the maturation of Antigen-Presenting Cells (APCs), such as Dendritic Cells (DCs) (61, 62). Immunological studies with ESAT-6, biochemically purified from short-term culture filtrates of Mtb, have highlighted its significance as a recognized antigen by T cells (16, 63–68). The adaptive immune response to ESAT-6 involves the activation of CD4+ and CD8+ T-cells (69). Studies have shown that ESAT-6-specific CD4+ T-cells produce IFN-γ and TNF-α, important cytokines in the control of Mtb infection (70). Furthermore, it has been observed that ESAT-6 directs Th17 cell differentiation by inducing IL-6 and TGF-β in DCs in a TLR-2- and MyD88-dependent manner (71). Hence, ESAT-6 may contribute to vaccine preparations by stimulating Th17 cell responses (71).

Several studies in mice have already been initiated following this perspective of ESAT-6 as a possible instrument, including promising results of a specific T-cell response to ESAT-6 with protective immunity comparable to that achieved with BCG (17, 72). However, these studies used different adjuvants to increase ESAT-6 immunogenicity (73), and the effects of this stimuli to human immune activation remains unclear. Future human studies are required to test the hypothesis of ESAT-6 as an inductor of protective immune responses (73). Thus, a few examples including the Cyclic Dimeric Adenosine Monophosphate (c-di-AMP) have been tested to potentiate the host humoral response against ESAT-6 (44). In mice, ESAT-6:c-di-AMP induces systemic Immunoglobulin (Ig) G and IgA secretion, demonstrating that c-di-AMP potentiates the host humoral response against ESAT-6 (74). Furthermore, ESAT-6:c-di-AMP induces a protective Th1/Th2/Th17 response and cytokine release in the spleen, whereas in the lung triggers Th17 and the differentiation of innate lymphoid cells (ILCs), mainly ILC3 (75). Thus, intranasal inoculation of ESAT-6:c-di-AMP promoted potential protective effects and it is a promising line of research (75). To expand the spectrum of immune responses, viral TB vaccines (namely TBvac-1, TBvac-2, and TBvac-10) have been developed, each encoding multiple established TB immunogens such as Ag85B, EsxH, and ESAT-6/EsxA. Notably, immunization with TBvac-1 or TBvac-2, both harboring the same trivalent antigens (Ag85B, EsxH, ESAT6/EsxA), resulted in a notable reduction of Mtb burden and limited lung tissue dissemination in a mouse model subjected to aerosol challenge [34]. Thus, the combination of ESAT-6/EsxA with Ag85B and EsxH enhances the generation of a long-term memory immune response (76). Thus, with the role of ESAT-6 as a pivotal antigen in TB vaccine design substantiated, and its conjunction with promising viral vectors like PICV established, novel vaccination strategies can be envisioned and developed. The crucial role of ESAT-6 in driving adaptive immune responses against Mtb highlights its significance for TB vaccine strategies and control efforts (77).

Despite the efficacy of BCG, the urgent demand for a vaccine specifically targeting pulmonary TB highlights the significance of assessing immune responses to Mtb antigens. This is particularly crucial since BCG effectiveness has been primarily demonstrated in infants and children, underscoring the gap in protection for adults and emphasizing the need for improved vaccine strategies that address the diverse manifestations and challenges of TB (78). Understanding ESAT-6 potential as a vaccine adjuvant and its impact on T cell activation and Th17 cell differentiation is crucial for developing effective TB vaccines, especially in endemic regions. Furthermore, clinical trials of ESAT-6 vaccination against TBwere completed in recent years, to test its efficacy as a vaccine subunit (79–83). Highlight, there is MTBVAC (84, 85), a vaccine that preserves the entire repertoire of T cell epitopes described for MTBC pathogens, including the main immunodominant antigens ESAT-6 and CFP-10. Currently, it is a strong candidate against TB in humans, as it has demonstrated great progress in phases 1 (84), and phase 2 (86). Thus, although some ESAT-6-basead vaccines have not presented clinical efficacy up to now, MTBVAC shows promise as it begins enrollment of phase 3 efficacy trials in infants born in sub-Saharan Africa [NCT04975178].

Nevertheless, the challenges associated with ESAT-6-based subunit vaccines are divided into two main pillars. The first is protective limitation, because although CD4 T cells are maintained in the lung parenchyma due to continuous antigenic stimulation, protective immunity is limited by functional exhaustion. The second, which would revolutionize the line of modern immunodiagnostics, is that an ESAT-6 vaccine would influencing in the well established tests (interferon gamma release assay (IGRA) and the tuberculin skin test (TST)), thus limiting the ability to distinguish immunized from infected people and the development of IGRA tests without ESAT-6 would be necessary (87). However, although the adaptive immunity developed by ESAT-6 is essential for the development of vaccines, during the latent phase of the disease it also favors the formation of the adaptive granuloma (88). This fact contributes to the immunopathology of TB, since the solid adaptive granuloma is a site of tissue damage in the early stages of the disease, as will be discussed in detail in section 4.2.

### 3.4 ESAT-6 as a biomarker

In addition to vaccine recognition epitopes, biomolecules such as ESAT-6 are emerging as promising biomarkers for diagnosis and immunotherapies. Most individuals infected with TB are known to remain asymptomatic, but around 5-10% of those infected progress to active TB within 5 years of first contact (89). Thus, diagnostic tests to screen for latent TB infection are essential to control active TB and consequently contain the spread of Mtb. IGRA and TST are the main methods for diagnosing TB infection nowadays (90).

The IGRA involves detecting IFN-γ levels post-stimulation by specific antigens of the pathogen, notably ESAT-6 and CFP-10 (91). Unlike the TST, IGRA focuses on RD1-encoded antigens absent in the BCG vaccine and most environmental mycobacteria, rendering it unaffected by BCG vaccination status or exposure to nontuberculous mycobacteria (NTM) (91). In newer-generation IGRA tests like QuantiFERON-TB Gold Plus (QFT-Plus), ESAT-6 and CFP-10 are designed to induce CD4+ T-specific responses alongside other peptides that stimulate both CD4+ and CD8+ T cells (92, 93).

Despite the effectiveness of many methods in diagnosing active TB, the high cost and time-consuming nature have led researchers to adopt more accurate and rapid screening methods based on Mtb-specific antigens. Recently, in order to ratify an effective and less costly diagnostic strategy, serum ESAT-6 levels in healthy people were compared with those of TB patients (94). The results showed TB patients had high levels of specific antibodies to the recombinant ESAT-6 and TST antigens if compared with healthy (non-TB) population (94). Thus, measuring antibodies against the recombinant ESAT-6 antigen, appears to be a possible diagnostic tool suitable for rapid and accurate screening of healthy and infected people.

In the current era of artificial intelligence, the development of predictive diagnostic models for differentiating active and asymptomatic TB has already begun (95). These models are based on specific and non-specific immunological indicators for TB, as well as multidimensional routine laboratory tests including routine blood tests, biochemistry, coagulation, and inflammatory reactions. Among these parameters, ESAT-6 was found to be decisive for the accurate performance of the created models (95). While initially developed within hospital settings, there is potential for broader application in community settings, suggesting that this algorithm could serve as a complementary tool for TB diagnosis. Moreover, ESAT-6 as a biomarker and the evaluation of its immune responses associated with Mtb in the host, may not only help in the diagnosis of TB, but may also be able to create lines of studies capable of predicting the immunopathogenic progression of the disease, favoring assertive interventions.

## 4 Cell death and TB pathogenesis

During Mtb infection, different modalities of host cell death occur, and the main ones are programmed cell death (PCD) and non-programmed cell death that contributes to the immunopathogenesis process of TB (96). Among the PCD processes, apoptosis is particularly notable due to its association with the containment strategy employed by mycobacteria. Conversely, necrosis, as a non-PCD pathway, facilitates the extracellular dissemination of the pathogen, eliciting acute inflammation through the release of cellular contents into the surrounding tissue (96). Over the years, more specific pathways of cellular necrosis that can be molecularly regulated have also been identified, including necroptosis, pyroptosis, and ferroptosis. It has been well-documented that ESAT-6 induces macrophage apoptosis (97). However, the impact of ESAT-6 on non-PCD pathways remains incompletely understood.

### 4.1 ESAT-6 and apoptosis: controlling the infection

Apoptosis is a conserved type of PCD regulated by caspases, which cleave cellular components to form membrane-bound apoptotic bodies, later phagocytosed by neighboring cells in a process called efferocytosis (96). Therefore, since Mtb is a pathogen that modulates autophagy and leverages the intracellular environment for its replication, the immune system uses the PCD as a method of containment. Thus, apoptosis is an important pathway to control Mtb infection (98). The main apoptotic pathways are subdivided in extrinsic (mediated by TNF-α/TNFR1 binding) and intrinsic (mediated by intracellular stresses that generate outer mitochondrial membrane permeability) (99). ESAT-6 is linked to apoptotic induction (100), but the specific molecular mechanisms involved in this process remain unclear. Nevertheless, it has been shown that this Mtb protein can activate the intrinsic pathway of apoptosis with increased levels of caspase-9 and -3 cleaved proteins (99). Upon ESAT-6 stimulation, Murine BMDMs exhibit Reactive Oxygen Species (ROS) production (101), Mitogen-activated protein kinases (MAPKs) activation (102), and increased cleaved of caspase-9 and -3 (99). Interestingly, when murine BMDMs cells are treated with antioxidants prior ESAT-6 stimulation, caspase-9 and -3 cleavage seem to be suppressed, suggesting that ESAT-6-induced ROS modulates the apoptotic response (99). ROS has been shown to act as second messengers and to promote pathological processes through the TLR2-dependent activation of MAPKs-capase-9/-3 cascade (102). Furthermore, miR-155 plays an important role in ESAT-6-mediated protective immune responses against Mtb. Upon ESAT-6 stimulation, macrophages exhibited increased expression of miR-155, leading to the activation of apoptotic pathways as evidenced by the activation of caspase-3 (61). Recently, the landscape screening of miRNAs induced by ESAT-6 showed that miR-431-3p and miR-1303 can both regulate macrophage apoptosis and phagocytosis (103). Interestingly, apoptosis miR-431-3P was found to modulate apoptotic pathway through the up-regulation of Multiple Drug Resistance type 1 (MDR1)/Matrix Metallopeptidase 16 (MMP16)/RHO Family Interacting Cell Polarization Regulator 2 (RIPOR2) genes and down-regulation ATG5 (103). Thus, the modulation apoptotic pathway represents a potential target for new therapeutic interventions aiming the control of Mtb infection as well as mitigating tissue damage.

### 4.2 Effects of ESAT-6 on cellular necrosis, granuloma formation and Mtb dissemination

Unlike apoptosis, necrosis is characterized by the disruption and leakage of plasma contents into tissue sites, causing inflammatory cascades and tissue damage and remodeling (104). Necrosis contributes to cellular injury and emerges from the formation of tuberculous granulomas, organized aggregates of macrophages recruited to contain the infection but also contributes to patient morbidity and Mtb dissemination (105), mainly through the manifestation of lung lesions. Of note, both non-necrotizing and necrotizing lesions cooperate to form an inflammatory superstructure that determines local immune responses (106). ESAT-6, particularly through the ESX-1 secretion system, promotes granuloma formation and the recruitment of macrophages to the infectious site (107). The dynamic nature of the granuloma in the lung serves as a complex hub where Mtb resides, while concurrently serving as a manifestation of the pathogen’s strategic maneuvers to ensure the prolonged survival of the host organism. Within this intricate milieu, a delicate interplay unfolds between pro-inflammatory microbicidal mechanisms and immunosuppressive processes. CD4+ effector T cells, including Th1 producing IFNγ and TNFα, Th17 secreting IL-17, and a smaller contingent of Th2 and regulatory T cells, orchestrate this balance (87, 108).

Interestingly, the necrotic death in human neutrophils has been reported to be linked to Mtb-induced ESAT-6-dependent calcium influx (109). ESAT-6 has also been shown to induce neutrophil extracellular traps (NETs) which influences chronic inflammation in Mtb infections through the upregulation of the cytokines IL-6, IL-1β and IL-10 (110). Furthermore, necrotic granuloma formation in zebrafish model of mycobacterial infection has also been reported to be associated with mTOR deficiency in an ESAT-6 dependent manner (109). Although ESAT-6 induces phagosomal damage independent of mTOR, this mycobacterial virulence determinant promotes mitochondrial damage that sensitizes cells to die through necrosis under mTOR deficiency conditions (109). Consequently, ESAT-6-induced mitochondrial damage is prevented by the presence of active mTOR, thus protecting cells to undergo death (109).

The role of ROS in driving neutrophil necrosis is also influenced by ESX-1, with ESAT-6 triggering ROS production and TNF activation, ultimately contributing to necrosis (111). Moreover, the interaction between ROS and TNF leads to mitochondrial damage, involving lysosomal proteases and the activation of BAX, which regulates mitochondrial Ca2+ influx through cyclophilin D (112). Pharmacological inhibition of L-type voltage-dependent Ca2+ channels (LTCCs) reduced Ca2+ uptake in macrophages and prevented mitochondrial overload, leading to the suppression of necrotic cell death (112). The inhibition of LTCCs also reduced necrosis via TNF, suggesting the possibility of suppressing ESAT-6-induced necrosis by preventing Ca2+ influx (113).

Modulating the apoptosis, the necrosis and macrophage activation with increased ROS production, that could lead to some mycobacterial killing, ESAT-6 contributes to the collateral tissue damage, the immunopathology. In addition, the cell death and stressed environment results in formation of DAMPs, amplifying the immune activation signals leading to a chronic process of inflammation and tissue damage.

## 5 Future perspectives

Understanding the pivotal role of ESAT-6 in the immunopathology of TB opens avenues for leveraging its potential in combating Mtb. ESAT-6 actively modulates the MyD88-dependent TLR signaling pathway, suggesting its utility as a tool for harnessing inhibitory mimetic peptides to regulate innate immune responses when prolonged TLR signaling proves detrimental. The negative regulation of autophagy by ESAT-6 is a critical factor in pathogen replication and disease propagation, hindering cellular recycling mechanisms. Consequently, the development of specific molecular tools targeting the inhibition of mTORC1 suppression and Mir-30a-5p could partially maintain homeostasis, preventing the activation of the NLRP3 inflammasome and mitigating TB severity and unfavorable treatment outcomes. Mainly because the formation of necrotic granuloma is associated with mTOR deficiency in an ESAT-6-dependent manner and mitochondrial damage is limited in the presence and activation of the mTOR complex, thus protecting from processes involving cell death.

Moreover, ESAT-6 potential within the adaptive response is noteworthy, with numerous studies in mice demonstrating its capacity to activate specific T cells with potential immune protective abilities. However, elucidating how ESAT-6’s protective capacity translates to humans warrants further investigation. While some studies have shown efficacy in using ESAT-6 as a vaccine adjuvant in rodents, this theory requires scrutiny in humans, necessitating the overcoming of key limitations, such as the functional exhaustion of ESAT-6-induced CD4 T cells and the development of new immunodiagnostic tests devoid of ESAT-6. Quantifying precise ESAT-6 thresholds within the human body to differentiate between infected and vaccinated individuals could refine diagnostic approaches. Additionally, utilizing ESAT-6 as a biomarker could complement diagnostics and predict immunopathogenic progression in positively reactive individuals. Understanding disease progression based on ESAT-6 levels at each stage of the infectious process could guide therapeutic interventions tailored to active TB cases and preemptively mitigate disease activation in latent infections.

Lastly, modulating apoptotic pathways to favor cell death before necrosis initiation through ESAT-6 holds promise, given its role in ROS activation essential for inflammatory imbalance in Mtb infections. Clarifying the specific molecular pathways involved in ESAT-6-induced apoptosis and understanding more clearily how ESAT-6 promotes macrophage recruitment to infectious sites via the ESX-1 secretion system are imperative. Additionally, it is suggested that, since the process of cell death through the activation of ESAT-6 involves the influx of calcium and, separately, the induction of cell death from the interaction of ROS and TNF is inhibited by inactivation of the type L Voltage-gated Ca2+ channels (LTCCs) reduced Ca2+ uptake in macrophages and prevented mitochondrial overload, it would be interesting to understand whether the cell death induction pathway by ESAT-6 could also be inhibited by inactivation of calcium channels.

Overall, unraveling ESAT-6’s multifaceted roles in TB immunopathogenesis presents promising avenues for therapeutic intervention and diagnostic advancement, underscoring its significance in combating Mtb.

## 6 Concluding remarks

Here, we highlight the role of ESAT-6 in TB immunopathology. The ESX-1/ESAT-6 secretion system is located at the RD1 locus, the region responsible for strengthening Mtb virulence. Through intricate metabolic pathways related to receptor activation, autophagy inhibition, and exacerbated cytokine production, ESAT-6 creates a conducive environment for Mtb survival within host cells. Consequently, the cellular perturbation caused by ESAT-6 can influence the fate of the host cell, modulating distinct modalities of cell death, determining the progression of the disease. Given the multifaceted implications of ESAT-6 in the regulation of TB pathogenesis, the pathways activated by this mycobacterial determinant hold significant potential as promising targets for the development of novel TB therapies. Furthermore, despite the initial portagonism of ESAT-6 in lysing membranes, we bring new studies that prove this fact clearly, rectifying that the potential for membrane disruption is attributed to the ESX-1 secretion system and not to ESAT-6. Notably, ESAT-6 is prominently recognized during early stages of Mtb infection and exhibits considerable immunogenicity, making it an attractive candidate for therapeutic intervention strategies. Understanding and effectively targeting the mechanisms orchestrated by ESAT-6 could lead to innovative therapeutic approaches that mitigate its detrimental effects on the host immune response and provide valuable tools to combat TB progression. As research in this area continues to evolve, harnessing the immunomodulatory properties of ESAT-6 may hold the key to advancing TB treatment and prevention strategies for better control of this global health burden.

## Author contributions

BP: Conceptualization, Data curation, Formal analysis, Investigation, Methodology, Visualization, Writing – original draft, Writing – review & editing. MA-P: Conceptualization, Formal analysis, Investigation, Visualization, Writing – original draft, Writing – review & editing. CV: Conceptualization, Investigation, Methodology, Visualization, Writing – original draft, Writing – review & editing. EA: Conceptualization, Supervision, Visualization, Writing – original draft, Writing – review & editing. BA: Conceptualization, Funding acquisition, Resources, Supervision, Visualization, Writing – original draft, Writing – review & editing.
